# Combined use of specific length amplified fragment sequencing (SLAF-seq) and bulked segregant analysis (BSA) for rapid identification of genes influencing fiber content of hemp (*Cannabis sativa L.*)

**DOI:** 10.1186/s12870-022-03594-w

**Published:** 2022-05-21

**Authors:** Yue Zhao, Yufeng Sun, Kun Cao, Xiaoyan Zhang, Jing Bian, Chengwei Han, Ying Jiang, Lei Xu, Xiaonan Wang

**Affiliations:** 1 Daqing Branch of Heilongjiang Academy of Sciences, Heilongjiang, China; 2 Daqing Branch of Heilongjiang Academy of Agricultural Sciences, Heilongjiang, China

**Keywords:** Hemp fiber content, Specific length amplified fragment sequencing, Bulked Segregant analysis, Candidate gene, SNP analysis

## Abstract

Hemp (*Cannabis sativa L.*), an ancient crop, is a significant source of high-quality fiber that primarily caters to the textile industry worldwide. Fiber content is a crucial quantitative trait for evaluating fiber yield in hemp. Understanding the genetic mechanisms involved in hemp breeding is essential for improving yield. In this study, we developed 660 F1 plants from a cross between Jindao-15 (high fiber content fiber-use variety) and Fire No.1 (low fiber content fiber-use variety), and thirty plants each with high and low fiber content were selected from 305 monoecious plants of this population according to 5%-10% of population size for quantitative traits. The DNA from these plants was extracted to establish two bulk DNA pools and then subjected to the restriction digestion by the enzymes RsaI and HaeIII to obtain 314–364 bp digestion fragments and subjected to sequencing using specific length amplified fragment sequencing (SLAF-seq). Finally, we successfully developed 368,404 SLAF tags, which led to the detection of 25,133 high-quality SNPs. Combing with the resequencing results of parents, the SNPs of mixed pools were then subjected to the SNP-Index correlation algorithm, which revealed four candidate regions related to fiber content traits on Chromosome 1, with a length of 8.68 Mb and containing 389 annotated genes. The annotation information and the comparison results identified 15 genes that were highly likely to modulate the fiber content of hemp. Further, qPCR validation identified six genes (LOC115705530, LOC115705875, LOC115704794, LOC115705371, LOC115705688 and LOC115707511) that were highly positively correlated with influencing the hemp fiber content. These genes were involved in the transcription regulation, auxin and water transportion, one carbon and sugar metabolism. And non-synnoumous mutation SNPs which may play vital role in influencing the fiber content were detected in LOC115705875, LOC115704794, LOC115705688 and LOC115707511. Thus, our study highlights the importance of the combined use of SLAF-Seq and Bulked Segregant analysis (BSA) to locate genes related to hemp fiber content rapidly. Hence, our study provides novel mechanistic inputs for the fast identification of genes related to important agronomic traits of hemp and other crops catering to the textile industry.

**Supplementary Information:**

The online version contains supplementary material available at 10.1186/s12870-022-03594-w.

## Introduction

*Cannabis sativa L*. is an annual herb that can be grouped as hemp or marijuana based on its tetrahydrocannabinol (THC) content, of which hemp contains less than 0.3% and marijuana contains more than 0.3% [1]. Traditionally hemp has been grown for its fiber and grain production. The hemp fiber has been used as raw material in ropes, fabric and sails fabrication. However, the safe, environmentally friendly, inexpensive and recyclable fiber raw material has been neglected for a long time due to its narcotic substance. Fortunately, the ability of remediation of polluted lands and fitting for rotation make this multi-purpose crop come back in the public view. Active research on hemp mainly focuses on determining the plant’s fiber, grain, and cannabidiol (CBD) content [2–4]. It is known that plant fibers have great potential to be used in various innovative applications as biodegradable, ecological, and renewable resources with unique properties [5, 6], with hemp bast fiber being one of the best [7]. Hemp bast fiber is the tissue outside the vascular cambium which located in the epidermis of the stalk. The fibers derived from the vascular bundles in the bast section contain about 20 mm to 50 mm long primary bast fibers and about 2 mm long secondary bast fibers. The other part of the stem was the core with rich lignin located in the ring of vascular cambium. The fiber of the core section is 0.5–0.6 mm long. [8–11] The primary bast fibers of hemp are made up of bundles of pericyclic elementary fibers that are characterized by thick and lignified cell walls. The main chemical composition of hemp bast fibers include about cellulose, pectins, lignin, hemicellulose, wax and ash. [12, 13] With the maturity of hemp, the cellulose content will increase continuously. The cellulose proportion can reach to 75% at the grain maturity. At the onset of flowering, the lignin content begin to increase while the hemicellulose one begin to decrease. The structure of cellulose was an intermediate between the highly crystalline cellulose in flax and semicrystalline one of kenaf [14]. The unique structure of hemp may explain its antibacterial effects [15], the ability to prevent damage due to exposure to radiation and ultraviolet rays [16], and the prevention of sound absorption and static [17]. Due to its capability to cater to military and civil needs, the primary bast fiber become an indispensable raw material in textile market. However, the price of hemp textiles is relatively high due to the production process and other factors. It is vital to increase the primary bast fiber output to meet the production demand and reduce the production cost.

It is reported that the probable measure to increase fiber yield through improving cultivation conditions [18, 19], but it has limited effects. Since a sole improvement of the hemp germplasm can fundamentally increase the yield, in this study, we investigate the crucial involvement of genetic factors in fiber production, apart from the roles played by the natural environment and the cultivation conditions. At present, hemp fiber improvement mainly relies on the traditional time-consuming method of phenotypic selection. Here, we highlight how delineating the molecular and genetic basis for hemp breeding can have an improved efficiency. However, it is imperative to identify the genes affecting hemp fiber yield to proceed with our proposed line of study. The current methods of gene mining mainly include reverse genetics approach and forward genetics one[20]. The development of sequencing techniques and bioinformatics has provided many valuable strategies for discovering new genes related to crop yields [21]. SLAF-seq combined with BSA is one of the positive genetics methods. Bulked segregant analysis (BSA) was first applied in plant genetics by Michelmore et al. [22]. All alleles must be present when DNA is bulked from a group of plants sharing the same phenotype. So the two bulked pools of segregating individuals differing for one trait will differ only at the locus that harbors that trait. Specific length amplified fragment sequencing (SLAF-seq) is a technique that uses high-depth sequencing to identify specific unique fragments in the genome and thereby help to locate molecular markers affecting the traits of interest. This method compares the differences in the SNP markers' occurrence frequency between two mixed pools with different genotypes. The advantages of such techniques include a high rate of identification of molecular marker density, low cost, high efficiency, and high accuracy [23]. The SLAF-seq technology has been implicated in multiple studies that include gene mapping of wheat, cucumber, pepper, melon, and other crops [24–27]. However, the use of SLAF-seq in determining hemp fiber yield remains largely unexplored. Fiber content has always been an important index to evaluate the hemp fiber yield, which is governed by multiple genetic components. It is known that the genetic component regulating hemp bast fiber content is significant, the gene-environment(G-E) interaction is low, and the generalized heritability is high [28]. No relevant studies have been reported to search for yield regulation genes from quantitative traits directly related to yield such as fiber content in hemp. However, related studies have been carried out on other fiber crops. Two ethylene pathway related genes related to yield have been found in cotton [29]. And a candidate gene that may control cotton lint percentage has been speculated through genome-wide association analysis [30]. The high heterozygosity and variability trigged by dioecious characteristic and out-crossing feature of hemp cause the separation of hemp traits in the F1 generation [30]. Laverty et al. have shown that the physical and genetic map of hemp can be constructed using the F1 generation population [31]. Such studies indicate the feasibility and importance of identifying genes regulating fiber yield by studying the quantitative traits of the F1 generation through SLAF and BSA technology.

This study used the isolated population constructed by crossing Jindao-15 and Fire No.1, which have a significant difference in their fiber content, to perform our investigations. We also coupled bioinformatic analysis to rapidly identify the genes regulating hemp fiber content with the SLAF-seq and BSA technologies. It greatly saved cost and improved gene mining efficiency. Our investigation provides a novel theoretical basis for improved breeding of hemp using molecular markers and genetic studies.

## Results

### Construction of mixed DNA pool

In F1 generation group, the highest fresh fiber content was 45.56%, and the lowest fiber content was 10.72% (Supplemental Table 1). From the F1 population, thirty plants with high fiber content (35.78%-45.56%) and thirty with low fiber content (10.72%-26.55%) were selected for further studies. An equal amount of genomic DNA was taken from each of the two groups and mixed to prepare a pool.

### Assessment result of construction of SLAF library using restriction digestion of the reference hemp genome

**Fig. 1:**
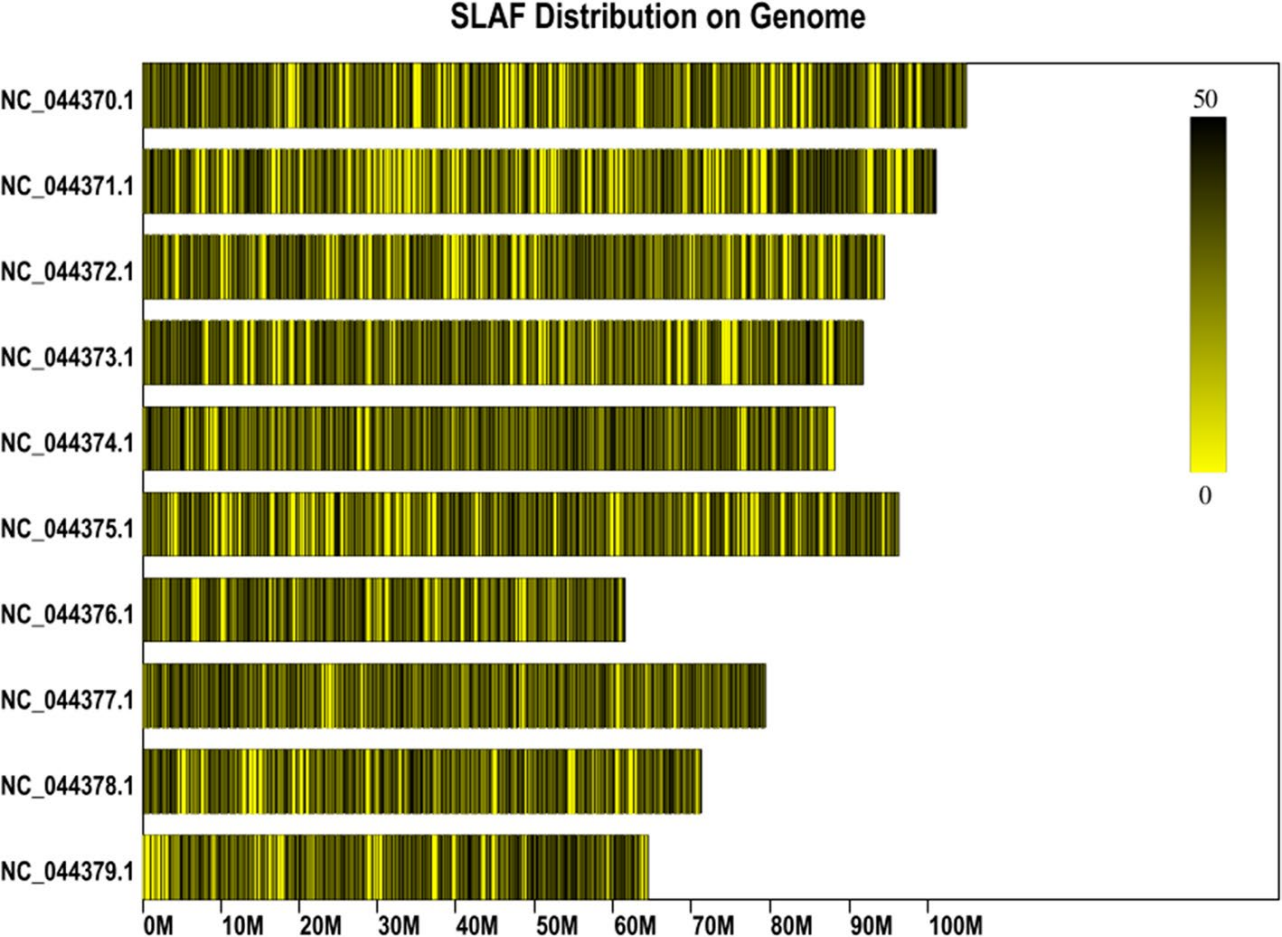
Distribution diagrams of all SLAFs (black lines) on each chromosome of hemp. The black lines indicate SLAF tags

It is already known that the size of the hemp reference genome is 876.148 Mb and the GC content is 34%. This led to the possibility of the formation of 105,823 SLAF tags from the hemp reference genome distributed uniformly on the chromosomes when subjected to restriction digestion (Fig. 1, Supplemental Table 2). Thus, the SLAF tag library was prepared by subjecting the DNA to RsaI and HaeIII mediated restriction digestion for further analysis.

## Identification of SNPs based on SLAF sequencing

Next, the sequencing reads obtained from each of the mixed pools were clustered for analysis. The clustered reads originating from the same SLAF fragment were defined as a particular SLAF tag. Each label, which showed differences in the sequence and SNPs between the different samples, was a polymorphic SLAF label. Our study identified 4,295,852 SNPs between the two parent populations, 389,687 SNPs from the bulked pools, and 102,964 polymorphic SLAF labels between the mixed pools (Supplemental Table 3). The results of the statistical analysis for SNP filtering are shown in Table 1. The comprehensive analysis finally identified 25,133 high-quality SNP sites in the hemp genome.

**Table 1:** SNP filtering statistic

Total SNP	Locus of multiple alleles	Locus with Read support less than 4	Locus of mixed pool genotypic uniformity	Locus filtered by the parent	High quality SNP
7,331,769	44,040	6,773,604	122,330	366,662	25,133

## SNP-index association analysis

**Fig. 2:**
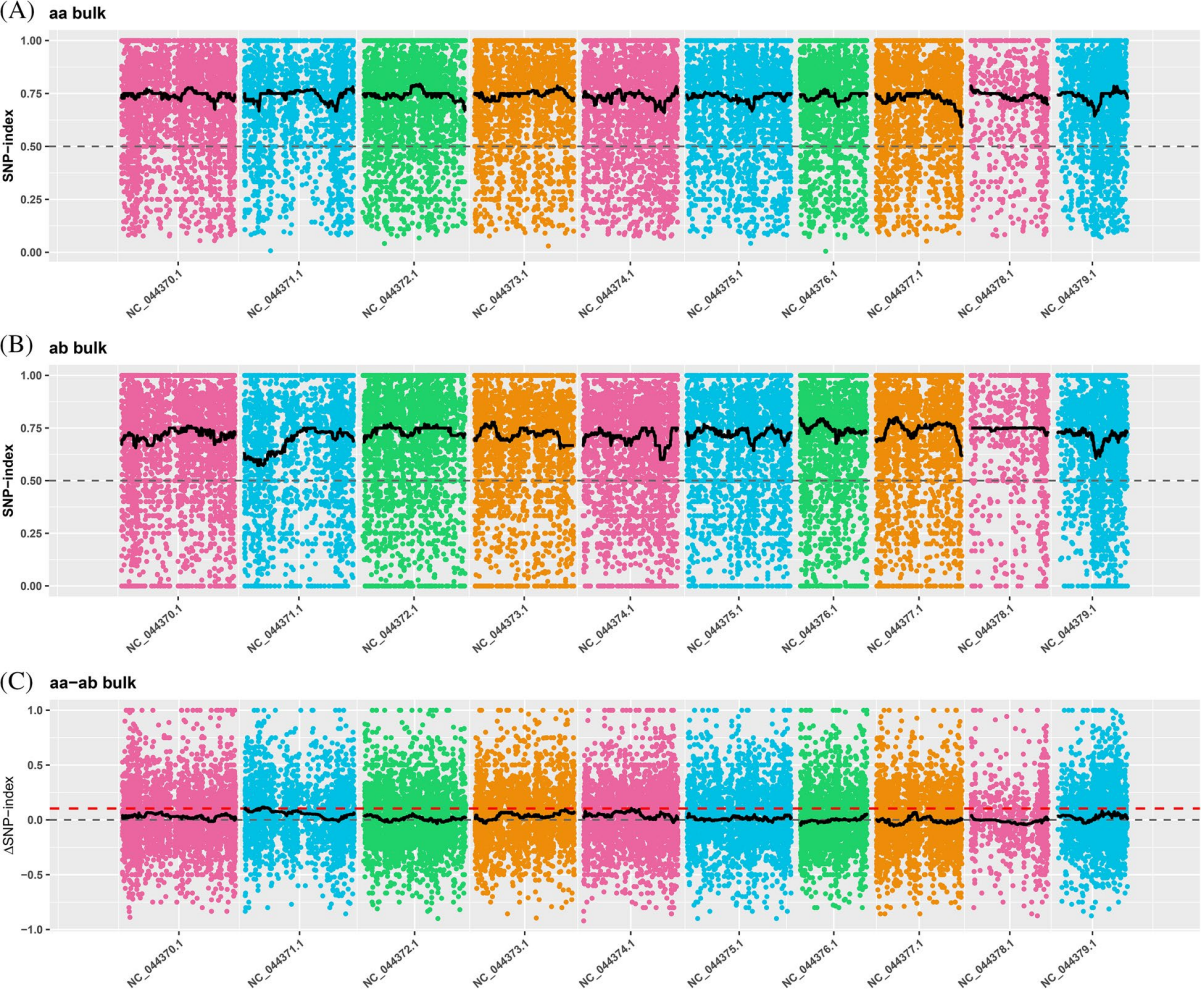
Distribution of SNP-index correlation values on chromosomes. The x-axis indicate chromosome name. Color dot represents the calculated SNPS—index (or Δ SNPS—index) values, the black line represents fitting SNP-index (or Δ SNPS—index) values. **A** SNP-index graphs of high fiber content mixed pool. **B** SNP-index graphs of low fiber content mixed pool. **C** Distribution diagram of ΔSNP-index value, of which the red dashed line represents the threshold line for the 99th percentile

Subsequently, a computer simulation experiment revealed no correlation with relevant candidate regions when the confidence was 0.90. Ideally, it is expected that the target site and its adjacent linkage sites should be close to the threshold, resulting in a high peak near the significant correlation region. However, due to the lack of regions exceeding the theoretical threshold, no significant position-related results were observed. To fully utilize the data, we lowered the theoretical threshold to locate possible areas of interest. We used the 99 percentile for fitting the Δ (SNP-index) (Fig. 2), namely the corresponding threshold 0.10, which revealed a total length of 8.68 Mb of four candidate regions on chromosome 1 of cs10 reference genome, including 397 genes. 12 genes were located in 12.34 Mb to 12.56 Mb interval. One gene was located in 14.32 Mb to 14.34 Mb interval. 336 genes were located in 14.60 Mb to 22.57 Mb interval. And 48 genes were located in 0.25 Mb to 0.72 Mb interval. The cultivar cs10 or ‘CBDRx' is a high-cannabinoid hemp type reference genome that is more closely-related to California marijuana than Asian fiber hemp, Finola or JL. Yet this reference genome information is more complete (with CDS files) compared to other published reference genomes [32]. The JL reference genome with CDS files was not published until 2020 [33] and therefore is not as well annotated.

## Association of regional gene annotation

**Fig. 3:**
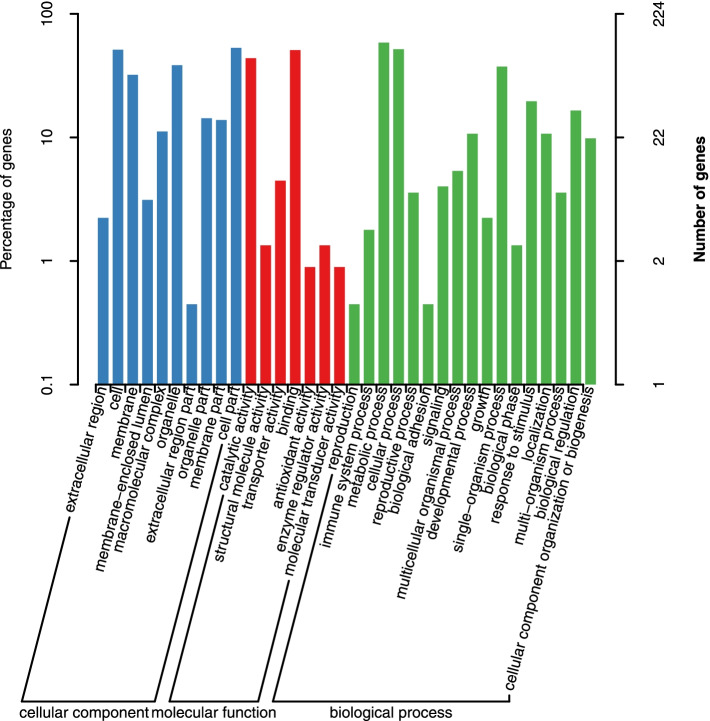
GO annotated results of genes in candidate regions

**Fig. 4:**
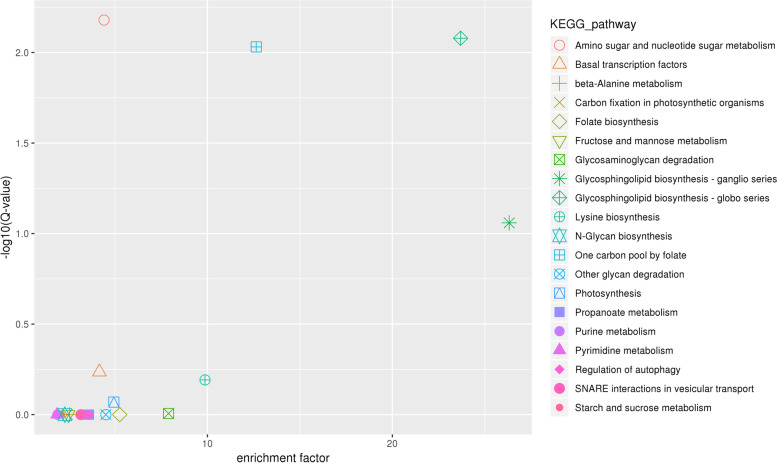
Genes enrichment results in the candidate regions via KEGG analysis [82–84]. The x-axis represents the enrichment factor and the y-axis represents Q-value. The smaller the Q-value, the higher the enrichment degree

Our study found that 389 genes encoded in the candidate regions identified from the previous analysis were annotated in multiple databases (Supplemental Table 4). 224 genes were identified through GO analysis (Fig. 3). Most genes are involved in biological process. KEGG analysis of the annotated genes from our study showed the enrichment of 142 identified genes closely associated with of amino sugar and nucleotide sugar metabolism, glycosphingolipid biosynthesis of both globo and ganglio series, one carbon pool by folate, basal transcription factors, lysine biosynthesis, photosynthesis, glycosaminoglycan degradation, and starch and sucrose metabolism pathways (Fig. 4).However, the involvement of these genes in affecting the hemp fiber content remains uninvestigated to date. A comparative homological analysis between the hemp genes and those of *Arabidopsis*, flax, and cotton genes and a thorough literature review helped us narrow down the scope of candidate genes. Finally, we chose 15 interesting candidate genes that might be involved in hemp fiber content regulation (LOC115705530, LOC115706200, LOC115707511, LOC115706733, LOC115704794, LOC115707202, LOC115707643, LOC115705371, LOC115705688, LOC115705010, LOC115705568, LOC115705891, LOC115706691, LOC115708167 and LOC115705875) for further analysis.

**Fig. 5:**
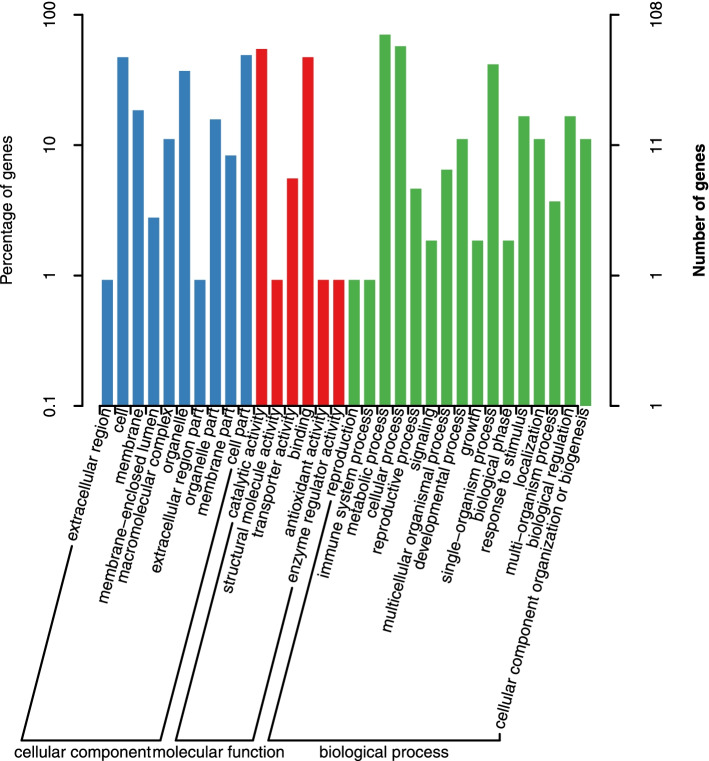
GO annotated results of non-synonymous genes

**Fig. 6:**
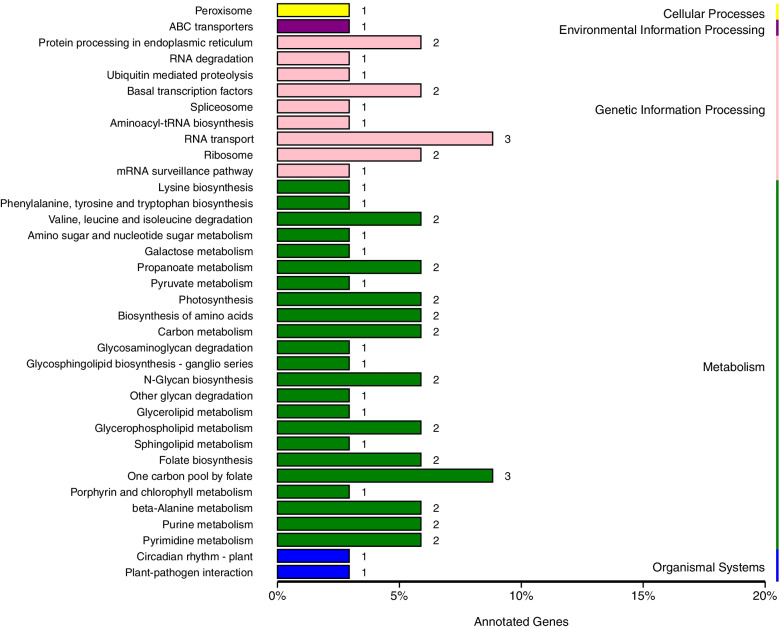
KEGG [82–84] annotated results of non-synonymous genes

SNPs can be either in the gene sequence or in non-coding sequences outside the gene. There are few SNPs located in protein coding region. There were 1938 SNPs in protein coding regions among parents and 178 SNPs in protein coding regions among mixed pools (Supplemental Table 5). Out of the total 389 annotated genes from the candidate regions, 199 harbored non-synonymous mutations between the parent populations and mixed pools, suggesting that a minimal number of these might play a functional role in regulating hemp fiber content. From these non-synonymous genes, 108 genes were annotated in GO database (Fig. 5) and 70 genes were annotated by KEGG analysis (Fig. 6). LOC115706200, LOC115707511, LOC115706733, LOC115704794, LOC115707202 and LOC115705688 have two to four non-synonymous SNPs in protein coding regions both in parents and mixed pools. Besides, LOC115706691, LOC115708167 and LOC115705875 have one to two non-synonymous SNPs in protein coding regions only among parents. These mutation SNPs may play a vital role in fiber content variation.

### Validation of Gene Expression by Real-Time quantitative PCR (RT-qPCR)

**Fig. 7:**
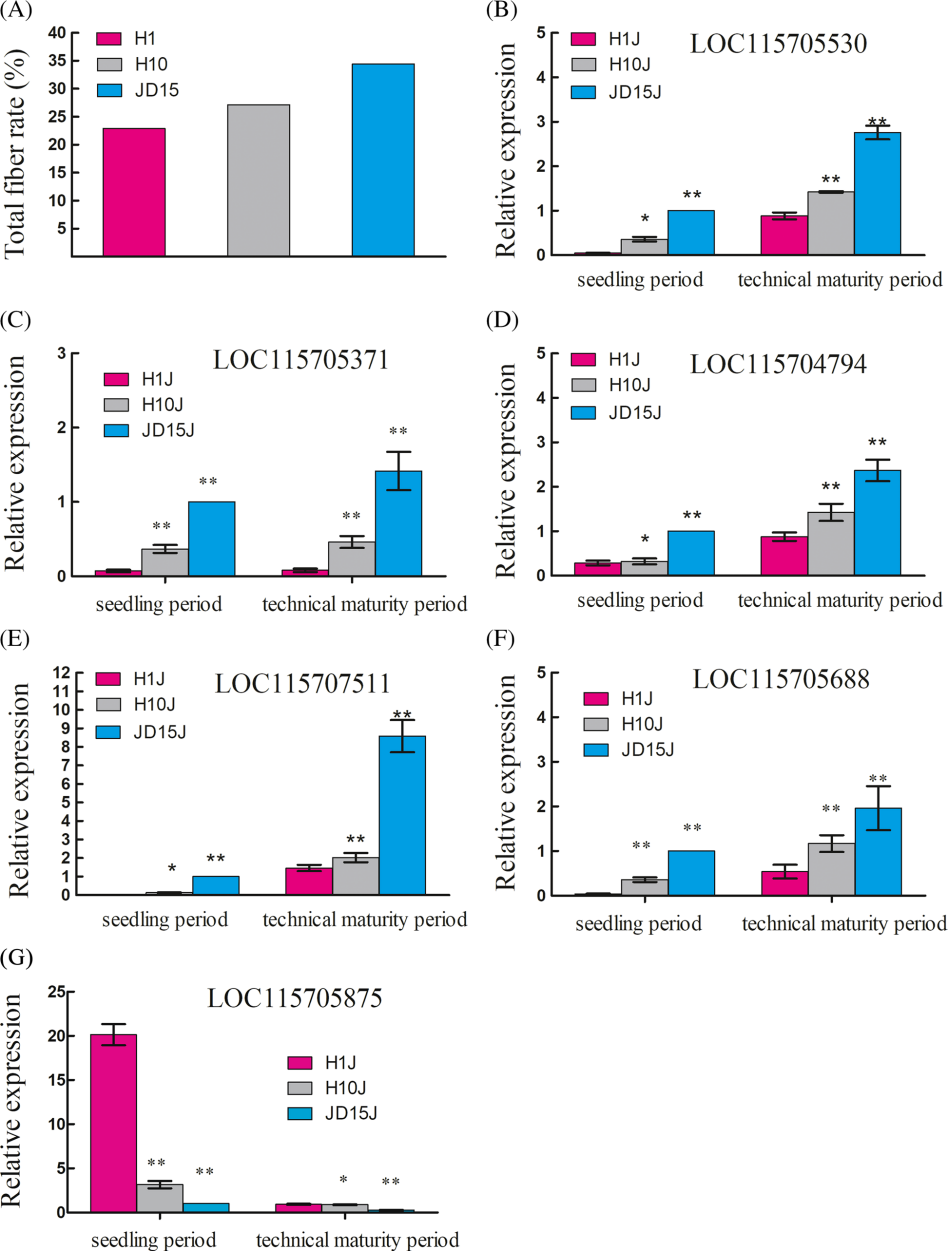
**A** The total fiber rate of three different hemp varieties. **B**-**G** Relative expression level of six candidate genes in different hemp varieties. Each bar represents the average expression level of three independent biological replicates. Error bars show standard errors of the average values. (*P < 0.05, **P < 0.01)

The stalks of Fire No. 1, Han Ma No. 10, and Jindao-15 were named H1J1, H10J1, and JD15J1, respectively, at the seedling period, while at maturity period, they were named H1J2, H10J2, and JD15J2, respectively. The gene expression analysis revealed an increase in the expression levels of transcription factor DIBARICATA (LOC115705530), WRKY DNA-binding transcription factor 70 (LOC115707511), aquaporin PIP1-2-like (LOC115704794), UDP-glucuronic acid decarboxylase 6 (LOC115705371), and bifunctional purine biosynthesis protein PurH (LOC115708688) with an increase in the total fiber content. In contrast, the expression levels of auxin transporter-like protein 2 (LOC115705875) decreased with the increase in total fiber content. The expression level of LOC115705875 in the seedling period was higher than that in the technical maturity period (Fig. 7G). The expression level of the five other genes in the seedling period did not increase to the extent of expression level in the technical maturity period (Fig. 7B-F). This analysis identified a differential expression pattern of the different candidate genes during the seedling and the mature phase, suggesting a growth phase-dependent involvement of multiple genes in regulating hemp fiber content.

## Discussion

Hemp fiber yield is a crucial parameter that affects the commercial production of textiles. It is challenging to identify the key genetic drivers regulating this complex yet essential trait. The genes regulating the fiber content of hemp remain unexplored to date. Hence, in this study, we leveraged the potential of next-generation sequencing (NGS), a highly efficient, cost-effective, and accurate method compared with conventional methods, to develop new genetic markers modulating essential traits such as fiber content of crops [34–36]. Recently, SLAF-seq has emerged as a technique with exceptionally high resolution and efficiency for identifying SNP markers in specific populations. Molecular markers can be directly developed from paired-end analysis of the sequence-specific restriction fragment lengths. Our study has identified 25,133 high-quality SNP locus that can be used to develop SNP molecular markers. Moreover, quantitative analysis showed that six genes (LOC115705530, LOC115705875, LOC115704794, LOC115705371, LOC115705688 and LOC115707511) may play essential role in hemp fiber content regulation.

One of the candidate genes identified by our analysis, LOC115705530, is located in 12.34 Mb to 12.56 Mb interval on chromosome 1. A comparative analysis revealed that this gene has the highest similarity with the gene At3G11280, which encodes the superfamily protein of replica-like homologous domain in *Arabidopsis thaliana*, and encodes for a protein that has a typical MYB domain. Studies involving the novel regulators of vascular development in *Arabidopsis thaliana* show that MYB has 40 interacting molecules [37]. Also, it has been reported that the gene FSM1, encoding an atypical MYB-like domain short protein found in tomato, negatively regulates the expansion of cells in the vascular bundle of fruit pericarp [38]. These studies collectively indicated an essential role of the MYB domain in regulating vascular development in multiple plants. MYB is also known to regulate lignin biosynthesis by recognizing AC elements in promoters of many lignin monomer biosynthesis genes [39] and is highly implicated in the regulation of secondary cell wall biosynthesis [40–42]. MYB, a transcription factor, is also closely associated with lignification in jute and ramie [43, 44], while MYB46-1, another MYB family transcription factor, regulates secondary cell wall and lignin biosynthesis in hemp [45]. However, further investigation is required to conclude the actual role of the gene identified in our study concerning hemp.

Another gene identified in our study was LOC115705875, located in 14.32 Mb to 14.34 Mb interval on chromosome 1, which had only one candidate gene. The protein encoded by this gene had the highest homology with AUXIN1 (AUX1). AUX1 had been reported to encode a high-affinity auxin influx vector. In *Arabidopsis thaliana*, AUX1 belongs to the AUX/LAX multigene family, consisting of four highly conserved genes AUX1 and Like AUX1 (LAX) genes LAX1, LAX2, and LAX3. All four AUX/LAX family members are known to have the auxin uptake function [46]. Auxin is an essential hormone for plant growth and development. Auxin flow carrier AUX1/LAX transports auxin into cells and promotes xylem differentiation in stem and root tissues by increasing cytoplasmic auxin signaling to regulate vascular patterns and differentiation [47].

Our RT-qPCR analysis showed that LOC115705875, the expression pattern of which shows a negative regulation during the seedling and the technical maturity period, differed in terms of function, with respect to the other genes mentioned. Low fiber-containing hemp varieties required more auxin, which could be attributed to the fact that a lower fiber content requires more auxin for xylem differentiation. The expression of this gene at the technical maturity stage was significantly lower than that at the seedling period, which might be because of the increased requirement of auxin by hemp during early development. Guerriero et al. found that the expression of genes related to auxin metabolism was higher in the older stem nodes at the 6-week seedling stage [48]. Studies on the secondary growth stage of hemp showed a high biological activity of auxin during the deposition and remodeling of the primary cell walls [49]. In other words, there is an increased demand for auxin in the early development of secondary phloem fibers of hemp. These reports collectively validate our findings based on the expression pattern of this gene and suggest a probable mechanism by which auxin might regulate the fiber content of hemp.

The next candidate gene, LOC115704794, was located in 14.60 Mb to 22.57 Mb interval on chromosome 1 and had the highest homology with AT4G00430, encoding aquaporin protein AtPIP1;4. Aquaporins(AQPs) are transmembrane channel proteins that regulate the intracellular and intercellular diffusion of water and other uncharged solutes such as glycerin, hydrogen peroxide, ammonia, small organic acids, urea, and metallic substances. AQPs are essential for maintaining water composition, osmotic regulation, signal transduction, detoxification processes, and the acquisition and transport of nutrients in various organisms [50, 51]. Plasma membrane intrinsic proteins (PIP) are one of five AQP subfamilies that have attracted particular attention for their potential to improve water retention and photosynthesis in plants [52]. PIPs can be divided into the subtypes PIP1 and PIP2 [53], which have an 80% amino acid sequence homology. The main differences between the two groups lie at the N- and C-terminal ends, the ring A length, and the amino acid composition [54]. The PIP1 subfamily was initially thought to be nonfunctional due to its failure to localize to the plasma membrane [55]. However, later experiments have shown that PIP1 does have a functional role inside the cell. For example, it was found that the stem parenchymal cells’ response to drought stress was significantly upregulated by the PIP1 subfamily of water channels, rather than the PIP2 subfamily [56].

Moreover, it is known that AtPIP1;2 in *Arabidopsis thaliana* promotes the water conductivity of roots and rosette leaves [57], while AtPIP1;4 mediates the transport of CO_2_, an essential regulator of photosynthesis [58]. However, a recent study has reported negligible changes in photosynthetic efficiency and mesophyll conductivity of *Arabidopsis* aquaporin knockout mutants (PIP1;2, PIP1;3, PIP2;6), compared to the control group [59]. A study based on cotton plants showed that GhPIP1-2, which belongs to the PIP1 family, is mainly expressed during the fiber extension period of cotton. The gene expression was recorded to be the highest at five days post-flowering, suggesting a vital role in supporting the rapid water flow into the vacuoles during cell elongation in cotton [60]. The transcriptional abundance of the PIP gene family in *Calotropis procera* fiber cells is greater than that of cotton. Studies on long thorns, which are adapted to survive in harsh environmental conditions such as drought and salty and alkaline conditions, also verified the role of PIP aquaporin in the elongation of fiber cells. However, the study suggests a more critical role of PIP2 than that of PIP1 [61]. Therefore, we conclude that LOC115704794 may affect the fiber yield by regulating the CO_2_ transport for photosynthesis. We also suggest a similar role of LOC115704794 to that of GhPIP1-2, whereby it adjusts the length and width of fibers to affect the fiber content. However, further experiments are needed to determine the exact mechanism by which LOC115704794 functions in regulating the hemp fiber content.

The following candidate gene, LOC115705371, also located in 14.60 Mb to 22.57 Mb interval on chromosome 1, had the highest similarity to the *Arabidopsis* gene AT2G28760, which encodes the protein UXS6. There are six UXS genes in the *Arabidopsis* genome, among which UXS3, UXS5, and UXS6 have the highest expression in the stem, mainly in the xylem cells and the interfascicular fibers. The proteins encoded by these genes, which regulate secondary wall formation, are directly regulated by the secondary wall NAC transcription factors. The simultaneous down-regulation/mutation of UXS3, UXS5, and UXS6 results in a significant decrease in the primary wall xyloglucan content, the thickening of the secondary wall, the content of xylan, and severe deformation of xylem vessels. Xylan and xyloglucan are the two main hemicelluloses in plant cell walls. Xylan is the main hemicellulose in the primary wall of dicotyledonous plants. Their biosynthesis requires a stable supply of sugar donors like UDP-xylose, which is synthesized by converting UDP-glucuronic acid via the activity of UDP-xylose synthase. UXS3, UXS5, and UXS6 play a significant role in the supply of UDP-xylose for the biosynthesis of xylan and xyloglucan [62]. Hence, we conclude that LOC115705371 may play a possible role in affecting the fiber content by regulating the hemicellulose content in hemp phloem fibers.

The following candidate gene, LOC115705688, located in 14.60 Mb to 22.57 Mb interval on chromosome 1, showed the highest similarity with AT2G35040. This gene encodes the protein phosphoribosylaminimidazole formamide formyltransferase, which belongs to the AICARFT/IMPCHase two-enzyme family proteins [63]. GO analysis showed that this gene was involved in nucleotide transport and metabolism, while KEGG pathway analysis showed the involvement of this gene in one-carbon (C1) metabolism. C1 metabolism is closely related to lignin biosynthesis [64–66]. Hemp lignification is one of the differentiation processes of bast fiber and core fiber. A comparative gene expression analysis of hemp bast fiber and core fiber showed that most of the coding proteins were involved in regulating C1 metabolism and lignin biosynthesis [67]. We conclude that LOC115705688 may participate in the lignification process to regulate hemp fiber content.

The final candidate gene, LOC115707511, located in 0.25 Mb to 0.72 Mb interval on chromosome 1, had the highest similarity with WRKY transcription factor WRKY70. WRKY transcription factors belong to one of the largest transcription factor families discovered to date and participate in regulating development, signal transduction, and stress defense processes in various plants. WRKY executes transcriptional activation or inhibition in either a homodimeric or a heterodimeric form [68]. WRKY70 has also been reported to regulate jasmonic acid and salicylic acid signaling [69]. A study of cotton plants showed that the laccase gene GhLac1 regulates fiber initiation and elongation by coordinating jasmonic acid and flavonoid metabolism [70]. WRKY transcription factors are also found to be up-regulated in jute during early fiber development [71]. The jasmonic acid biosynthesis-related gene expression in adult stem nodes was higher in the phloem [72]. Therefore, we conclude collectively from all the studies that WRKY70 may affect fiber development by regulating the jasmonic acid pathway.

No SNP was dectected in LOC115705530. The SNP mutation of LOC115705875 made Glycine change into Alanine. In LOC115704794, Alanine, Leucme and Glutamine were transformed into Proline, Valine and Lysine respectively. No coding SNP were found in LOC115705371. Four SNPs sites mutation lead the coding amino acid Alanine, Proline, Asparticacid and Arginine change into Threonine, Serine, Glycine and Tryptophan in LOC115705688. Leucine and Serine varied into Isoleucine and Threonine in LOC115707511. These amino acids mutation may lead to the variation of related protein structure and function. More research is needed to understand how these non-synonymous mutation affect the involvement protein function by altering the protein structure.

Our study highlights our novel identification of genes involved in regulating the fiber content trait of hemp by using the integrated SLAF-seq and BSA methods. The findings of our study have the potential to lay a good foundation for determining regulators of hemp breeding via molecular marker-assisted selection. The advantage of using an F_1_ population to identify fiber content related genes is saving time and effort. However, each genotype is not possible to measure repeatedly. Therefore, this method is suitable for detecting major quantitative trait gene locus. The accuracy of target gene location determining the essential agronomy trait can be improved by combination of multiple post-genomics techniques such as transcriptomics, proteomics and metabolomics. It has been reported that some genes related to fiber development have been detected through transcriptome analysis of hemp bast fiber at different stages. The integration of existing results may promote the process in discovery of key genes regulating agronomy trait. And it is more persuasive to identify the candidate genes taking advantage of diverse genotype germplasms. Moreover, further in-depth analysis and functional characterization of these candidate genes by transformation or assessment of mutation are required to delineate their roles in regulating hemp fiber content conclusively.

## Conclusions

In our study, the F1 population was constructed by crossbreeding hemp parents with a significant difference in total fiber content. The mixing pool was established according to the fiber content, and SLAF database construction and sequencing were performed. A total of 368,404 SLAF tags were developed, and 25,133 high-quality SNP sites were detected. According to the SNP-Index correlation algorithm, four candidate regions related to fiber content traits were obtained on chromosome 1, with a length of 8.68 Mb and 389 annotated genes, among which 199 genes were non-synonymous mutants between the parental populations and mixed pools. According to the annotation and comparison results, the 15 genes (LOC115705530, LOC115706200, LOC115707511, LOC115706733, LOC115704794, LOC115707202, LOC115707643, LOC115705371, LOC115705688, LOC115705010, LOC115705568, LOC115705891, LOC115706691, LOC115708167 and LOC115705875) were highly significantly positively correlated candidate genes for hemp fiber content. The quantitative analysis validated that the genes LOC115705530, LOC115705875, LOC115704794, LOC115705371, LOC115705688 and LOC115707511 were indeed positively correlated with fiber content. And non-synonymous mutation SNPs which may play vital role in influencing the fiber content were detected in LOC115705875, LOC115704794, LOC115705688 and LOC115707511. These studies indicated that SLAF-Seq and BSA methods could be used to locate genes related to important agronomic traits in hemp rapidly.

## Materials and methods

### Plant materials used and construction of two distinct pools of plants

**Fig. 8:**
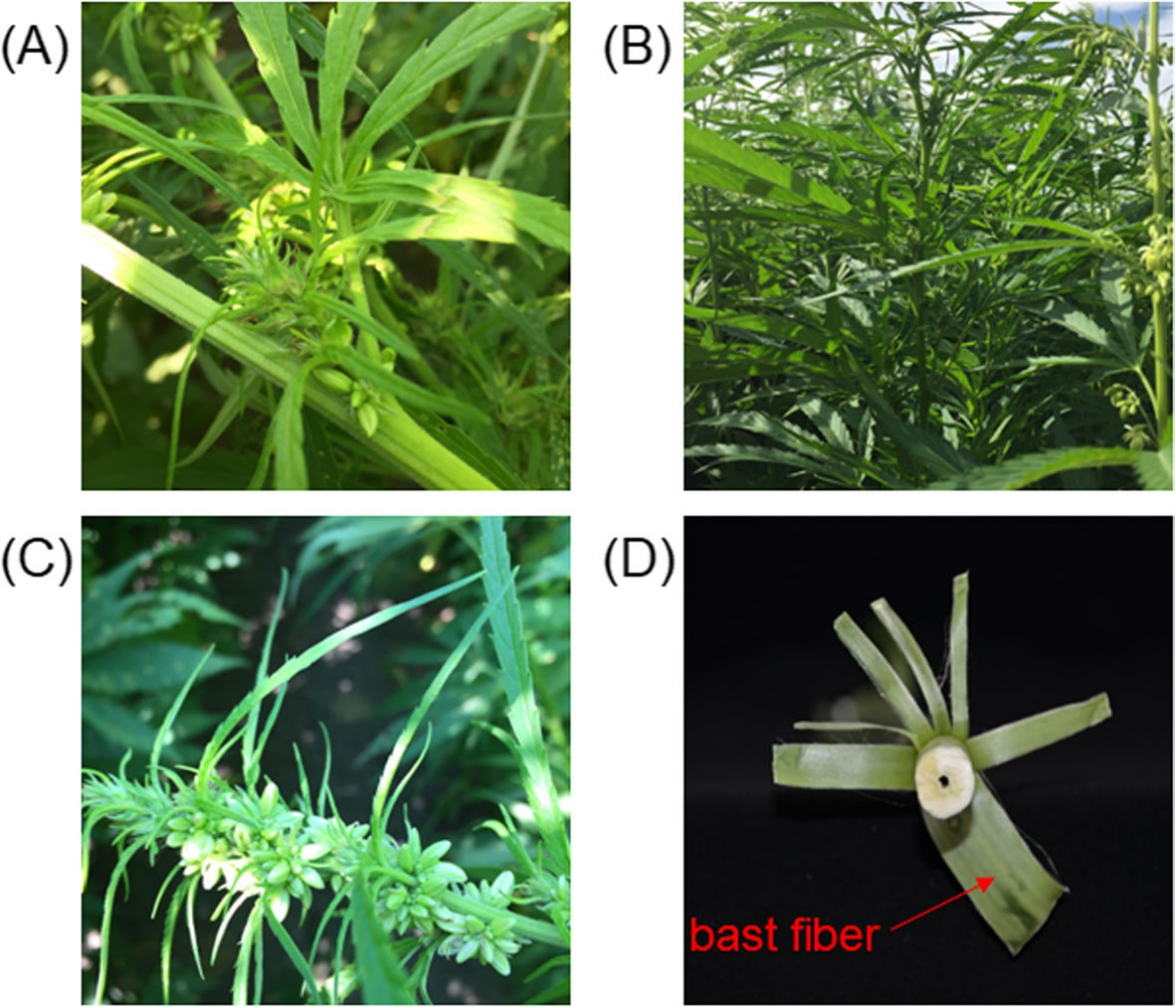
Experimental cultivars and materials **A** Monoecious Jindao-15. **B** Female plant of dioecious Fire No.1 **C** Male plant of Fire No.1.**D** Fresh bast fiber peeled from stem

The selection of plant varieties for this study depended on the total fiber rate (calculated after retting) which can indicate the fiber content. Jindao-15 (Fig. 8A)was produced in the Ukrainian Academy of Agricultural Sciences in 2014, and its total fiber rate is 34.4% [73]. Fire No.1 (Fig. 8B, C) was bred by Daqing Branch of Heilongjiang Academy of Sciences in 2015, with a total fiber rate of 22.9% [73]. These cultivars were provided by the Daqing Branch of Heilongjiang Academy of Sciences. Jindao-15 and Fire No.1 were crossed as the male and female parents respectively to achieve the F1 generation group. The monoecious hemp cultivar Jindao-15 and the dioecious Fire No.1 were grown in the greenhouse of Chunlei farm (Daqing, Heilongjiang, China) in the winter, 2018. The male plants of Fire No.1 were uprooted when the buds appeared. A total of 660 seeds were obtained by crossing the two cultivars in the spring of 2019. The seeds and their parents were then grown in an experimental base of Dongfeng farm under natural conditions. The leaves of 305 monoecious plants of the F1 population were collected and immediately immersed into liquid nitrogen during budding. The leaves were stored at -80˚C. The 305 plants were harvested during the initiation of flowering. The weights of fresh plant stems and bast fibers (Fig. 8D) peeled from the stem were recorded. Phenotypic data were statistically analyzed to calculate the fiber content (Fiber content = bast fiber weight/ stem weight × 100%) using Microsoft Excel (Microsoft Office, Microsoft, 2003). Thirty of each high and low fiber-containing plants were selected for the extreme bulk construction. Total genomic DNA was isolated from tender leaves of both parental lines and every plant of the two segregating bulks using a Plant Genomic DNA Kit (Tiangen Biotech, Beijing, China) following the manufacturer’s protocol. DNA concentration and quality were measured by 1% agarose gel electrophoresis. Thus, considering equal amounts of DNA from thirty high fiber-containing plants and thirty low fiber-containing plants, two separate and distinct gene pools were made.

### Construction of SLAF-seq library and high-throughput sequencing

The entire genome of hemp (*Cannabis_sativa* L.) (https://www.ncbi.nlm.nih.gov/assembly/GCF_900626175.1/) was chosen as the reference genome for this study. The SLAF-predict v2.0 software (provided by Biomarker Technologies Corporation) was used to predict the digestion pattern of the hemp reference genome using restriction endonucleases. First, electronic digestion simulation was carried out to analyse the appropriate enzyme combination amount according to the reference genome. In this step, the proportion of repeats sequences in enzyme digestion combinations and coverage of gene regions were calculated to avoid the known repeated sequences. Next, in every candidate combination, the situation of each position and length of each fragment was simulated in detail, and the stability and redundancy of the number of film segments were evaluated. Based on above analysis, RsaI and HaeIII were chosen to get the SLAF tag fragments of length 314 ~ 364 bp for SLAF library construction. Single-nucleotide A overhangs and dual-index sequencing adaptors were ligated to these fragments obtained by digestion of the hemp genomic DNA [74]. The modified fragments were then amplified by PCR, purified, pooled, and screened to construct the SLAF library. A detailed description of SLAF library construction and screening processes is given by Sun et al. [23]. Finally, the SLAF library was sequenced at an Illumina HiSeq 2500 platform (Illumina, Inc., San Diego, CA, USA) at Biomarker Technologies Corporation in Beijing. The Oryza sativa genome(http://www.ncbi.nlm.nih.gov/genome/?term=Arabidopsis%20thaliana, version 7.0) was used as a positive control to evaluate the quality of the experiment and the enzyme digestion reaction. The control sequencing reads were compared with the reference genome by Burrows-Wheeler Alignment tool (BWA)software(https://nchc.dl.sourceforge.net/project/bio-bwa/bwa-0.7.15.tar.bz2).

Our analysis led to the generation of 36.45 Gb clean data developed from the parents through resequencing, with the average sequencing depth of the individual populations being 10.40 X. The sequencing data associated with this study have been deposited in NCBI with accession number PRJNA749899. (https://www.ncbi.nlm.nih.gov/bioproject/?term=PRJNA749899).To further characterize the genetic elements affecting hemp fiber production, we developed 368,404 SLAF tags from the DNA pool created by mixing the two populations using the simplified SLAF genome sequencing method (Supplemental Table 3).

An average Q30 (Q30 indicates a quality score of 30, indicating a 0.1% chance of error and thus 99.9% confidence) ratio of 93.41% and 37.94% GC content (Table 2) revealed the number of reads and the quality of the data, respectively, from the sequencing analysis. The numbers of SLAF tags in the high and low fiber-containing pools were 225,318 and 320,016, respectively. Moreover, the sequencing depth of the former pool was 41.66X, while that of the latter was 48.19X. BWA software analysis of the sequencing reads showed a 90.88% efficiency (within the normal range) of the double end analysis compared to the reference rice genome. We also observed that the actual lengths of the SLAF tags calculated according to the pair-end mapped reads of contrast locations in the genome correspond to the expected fragment size.

**Table 2:** Statistic results of sequencing data from each sample

Sample	Sample ID	Clean-Read number	Clean-Base number	Q30 percentage(%)	GC percentage(%)
Male parent	R01	61,937,343	18,556,453,868	93.05	35.00
Female parent	R02	59,738,133	17,895,069,796	93.60	34.16
High-fiber content pool	aa	12,354,037	3,113,217,324	93.40	42.51
Low-fiber content pool	ab	19,527,628	4,920,962,256	93.60	40.08

## Identification of high-quality SNPs

To ensure high quality of data, low-quality raw reads and reads with adapter were filtered to retain only the clean reads. Low-quality raw reads include the reads which have more than 10% undetermined base types in one read and the reads which number of bases with quality value Q ≤ 10 accounts for more than 50% of the entire read. The BWA software was used to compare the clean reads with the reference genome to locate their positions. The samples' sequencing depth, genome coverage, and other crucial information were recorded, followed by detection of variations.

According to the localization of the clean reads in the reference genome, the Mark Duplicate tool of Picard (http://sourceforge.net/projects/picard/.) was used to remove duplicates to negate the impact of PCR duplication. Base recalibration and variant calling were performed using Genome Analysis Toolkit (GATK) software (https://gatk.broadinstitute.org/hc/en-us) [75]. The SNPs were filtered strictly to get the final set of SNPs. First, SNPs with multiple genotypes were filtered out, then SNPs with read support less than four were filtered out, and then SNPs with consistent genotypes between mixing pools and SNPs with recessive mixing pool genes that did not come from recessive parents were filtered out. At last, the SNPs with QUAL < 30, QD < 2.0, FS > 60.0 and MQ < 40.0 were filtered. All the variable sites between the test and the reference genomes were identified based on a comparison between the two. All the variations in SNP annotation and effectiveness prediction were identified using the SnpEff software (https://sourceforge.net/projects/snpeff/).

## Association analysis

SNP-index is a marker correlation analysis method that relies on the differences in the genotype frequency between the mixing pools [76, 77]. This analysis mainly looks for significant differences in genotype frequency between the mixed pools with Δ (SNP-index) statistics. The closer a marker is associated with a trait, the closer the Δ (SNP-index) value reaches. The supplemental table 6 shows the calculation formulae.

The process of elimination of false-positive sites mainly involves the identification of different markers located in the genome. The SNPNUM method (a script written by Biomarker Technologies Corporation) was adopted to plot the Δ (SNP-index) values. The target locus and its adjacent linkage loci should approach the threshold which normally is 99% when a high peak should occur near the significantly associated region. The result was calculated by running a script developed by Biomarker Technologies Corporation based on Permutation test principle. A sliding window value m was obtained by taking an average of Δ(SNP-index) of 10 consecutive SNPs. This process was simulated 10,000 replications to obtain null distribution (SNPs that are not selected) of m for F1 generation. The detailed method was described by the study of Takagi. [78].

### Gene annotation

The coding genes in the candidate area of confidence interval were accurately annotated using Non-redundant(NR) [79], Swiss-prot [80], Gene Ontology (GO) [81], Kyoto Encyclopedia of Genes and Genomes (KEGG) [82–84], and Cluster of Orthologous Groups of proteins (COG) [85] by BLAST software [86]. Such detailed annotation helps in the rapid screening of candidate genes.

### Validation of Gene Expression by Real-Time quantitative PCR (qPCR)

The expression levels of candidate genes were validated by the Real-time quantitative PCR (qPCR) method. Total RNA was extracted from stalks of 3 varieties of hemp with different total fiber rates (Fig. 7A) (Fire No.1 (22.9%), Han Ma No.10 (27.1%), and Jindao-15 (34.4%)) at the seedling stage and the technical maturity stage using RNA Extraction Kit (Tiangen, China). The RNA was dissolved in Ultra Pure™ DNase/RNase-free distilled water (Invitrogen, USA). The total RNA was reverse-transcribed using FastKing RT Kit (KR116) reagent. Primer sequences were designed using Primer 7.0 and screened using SeqHunter 1.0 (Supplemental Table 7). The qPCRs were performed using Power qPCR PreMix (Genecopoeia, USA) according to the manufacturer’s instructions. SYBR Green PCR cycling was performed in an IQTM5 Multicolor Real-Time PCR Detection System (Bio-Rad, USA) using 20 μl reaction volumes. The reaction conditions are as follows: 95 ℃ for 10 min, followed by 40 cycles of 95 ℃ for 10 s, 60 ℃ for 40 s, and 60 ℃ for 15 s. The relative quantitation of gene expression was calculated and normalized to glyceraldehyde-3-phosphate dehydrogenase (GAPDH), used as a housekeeping gene. Three biological replicates from each condition were used for the qPCRs.

## Supplementary Information

**Additional file 1:** **Supplemental Table 1.** Detailed information about the fresh fiber content of F1 bulk and mixed pool

**Additional file 2:** **Supplemental Table 2.** Numberof all SLAFs on each chromosome

**Additional file 3: Supplemental Table 3. **The statistical result of SLAF tags and polymorphic SLAF tags 

**Additional file 4:** **Supplemental Table 4.** The statistical analysis of annotated genes in the associated regions

**Additional file 5: Supplemental Table 5. **Theresult of the SNPs annotation classification 

**Additional file 6:** **Supplemental Table 6.** SNP-index Calculation formulae

**Additional file 7: Supplemental Table 7. **Primer sequences were used for Real-Time quantitative PCR validation 

## Data Availability

All data generated or analysed during this study are included in this published article. And the sequencing data that support the findings of this study are available from NCBI (https://www.ncbi.nlm.nih.gov/bioproject/?term=PRJNA749899) under accession number PRJNA749899.

## Abbreviations

SLAF-seq: Specific Length Amplified Fragment Sequencing
BSA: Bulked Segregant Analysis
SNP: Single nucleotide polymorphism
GO: GeneOntology
KEGG: Kyoto Encyclopedia of Genes and Genomes
RT-qPCR: Real-time quantitative PCR
GAPDH: Glyceraldehyde-3-phosphate dehydrogenase
