# Supplementary material for: Combined use of specific length amplified fragment sequencing (SLAF-seq) and bulked segregant analysis (BSA) for rapid identification of genes influencing fiber content of hemp (Cannabis sativa L.)
Source: BMC Plant Biol. 2022 May 21;22:250. doi: 10.1186/s12870-022-03594-w (PMC9123736; doi:10.1186/s12870-022-03594-w)
Supplement: Supplementary file 2 — Additional file 2: Supplemental Table 2. Numberof all SLAFs on each chromosome [file 12870_2022_3594_MOESM2_ESM.doc]

Supplemental Table 2 Number of all SLAFs on each chromosome

| Chromosome_ID | Chromosome_length | Expected_SLAF_number | Average_SLAF_distance |
| --- | --- | --- | --- |
| NC_044370.1 | 104987320 | 13010.7 | 8069.31 |
| NC_044371.1 | 101209240 | 11194.1 | 9041.34 |
| NC_044372.1 | 94670641 | 11249.6 | 8415.47 |
| NC_044373.1 | 91913879 | 11419.3 | 8048.97 |
| NC_044374.1 | 88181582 | 11734.1 | 7514.98 |
| NC_044375.1 | 96346938 | 11349.4 | 8489.18 |
| NC_044376.1 | 61561104 | 7909.49 | 7783.19 |
| NC_044377.1 | 79335105 | 10857.7 | 7306.82 |
| NC_044378.1 | 71238074 | 8857.93 | 8042.29 |
| NC_044379.1 | 64622176 | 8240.72 | 7841.81 |
| Total | 854066059 | 105823 | 8070.7 |
