# Supplementary material for: Combined use of specific length amplified fragment sequencing (SLAF-seq) and bulked segregant analysis (BSA) for rapid identification of genes influencing fiber content of hemp (Cannabis sativa L.)
Source: BMC Plant Biol. 2022 May 21;22:250. doi: 10.1186/s12870-022-03594-w (PMC9123736; doi:10.1186/s12870-022-03594-w)
Supplement: Supplementary file 5 — Additional file 5: Supplemental Table 5. Theresult of the SNPs annotation classification [file 12870_2022_3594_MOESM5_ESM.doc]

Supplemental Table 5 The result of the SNPs annotation classification

| Type | R01vsR02 | aavsab |
| --- | --- | --- |
| UTR_5_prime | 222 | 11 |
| UTR_3_prime | 332 | 24 |
| Upstream | 6,571 | 433 |
| Synonymous-stop | 1 | 0 |
| Synonymous-coding | 1,130 | 96 |
| Stop-lost | 6 | 0 |
| Stop-gained | 30 | 2 |
| Start_lost | 2 | 0 |
| Start_gained | 28 | 0 |
| Splice-site-region | 88 | 7 |
| Splice-site-donor | 1 | 0 |
| Splice-site-acceptor | 4 | 0 |
| Non-synonymous-coding | 808 | 82 |
| Intron | 3,578 | 285 |
| Intergenic | 14,310 | 1,469 |
| Downstream | 5,296 | 467 |
| Other | 3 | 2 |
