# Supplementary material for: Combined use of specific length amplified fragment sequencing (SLAF-seq) and bulked segregant analysis (BSA) for rapid identification of genes influencing fiber content of hemp (Cannabis sativa L.)
Source: BMC Plant Biol. 2022 May 21;22:250. doi: 10.1186/s12870-022-03594-w (PMC9123736; doi:10.1186/s12870-022-03594-w)
Supplement: Supplementary file 6 — Additional file 6: Supplemental Table 6. SNP-index Calculation formulae [file 12870_2022_3594_MOESM6_ESM.doc]

Supplemental Table 6 SNP-index Calculation formulae

| Calculation formulae |
| --- |
| SNPindex（aa）=Maa/(Maa＋Paa)  Maa showed the female sample depth from aa pool while Paa indicate the male sample depth from the same pool. |
| SNPindex（ab）=Mab/(Mab＋Pab)  Mab stood for the female sample depth from ab pool while Pab was the male sample depth from the same pool |
| Δ(SNP-index) = SNPindex（aa）–SNPindex（ab） |
